# Invited Article: A precise instrument to determine the Planck constant, and the future kilogram

**DOI:** 10.1063/1.4953825

**Published:** 2016-06

**Authors:** D. Haddad, F. Seifert, L. S. Chao, S. Li, D. B. Newell, J. R. Pratt, C. Williams, S. Schlamminger

**Affiliations:** 1 National Institute of Standards and Technology (NIST), 100 Bureau Drive Stop 8171, Gaithersburg, Maryland 20899, USA; 2 University of Maryland, Joint Quantum Institute, College Park, Maryland 20742, USA

## Abstract

A precise instrument, called a watt balance, compares mechanical power measured in terms of the meter, the second, and the kilogram to electrical power measured in terms of the volt and the ohm. A direct link between mechanical action and the Planck constant is established by the practical realization of the electrical units derived from the Josephson and the quantum Hall effects. We describe in this paper the fourth-generation watt balance at the National Institute of Standards and Technology (NIST), and report our initial determination of the Planck constant obtained from data taken in late 2015 and the beginning of 2016. A comprehensive analysis of the data and the associated uncertainties led to the SI value of the Planck constant, *h* = 6.626 069 83(22) × 10^−34^ J s. The relative standard uncertainty associated with this result is 34 × 10^−9^.

## I. BACKGROUND ON WATT BALANCES AND THE LINK TO *h*

The watt balance is both an instrument and an experiment, and has its origins in the earliest efforts to create a fundamental electrical standard for the ampere, the base unit of electrical current in the international system of units (SI).^1^ It helps to recall the SI definition of the ampere: “The ampere is that constant current which, if maintained in two straight parallel conductors of infinite length, of a negligible circular cross section, and placed 1 meter apart in a vacuum, would produce between these conductors a force equal to 2 × 10^−7^ N/m of length.” This base electrical unit is defined in terms of a mechanical quantity, force, which must be measured using standards of length, time, and mass, just as originally suggested by Maxwell.^2^ Ampere balance experiments, and eventually watt balance experiments, were conceived as a means of carrying out such measurements using a mechanical balance to compare an electromagnetic force produced by a coil in a magnetic field to the weight of a test mass, i.e.,

(1) $$F_{z}=mg=-I\frac{\partial \Phi }{\partial z},$$

where *m* is the mass of the test mass, *g* the local gravitational acceleration, *I* the current in the coil, and Φ the magnetic flux through the coil. The point of the experiment is to derive the value of *I* in terms of the other quantities, which are mechanical in nature. In the original ampere balances, the flux was produced by a second coil. The calculation of the flux from this coil, which is the integral of the magnetic flux density ***B*** over the area segment d***A***, required absolute measurement of the coil physical dimensions, which was difficult and limited the precision that could be achieved. In 1976, Kibble^3^ published a fundamental insight that significantly changed the field: The vertical derivative of the magnetic flux could be calculated as the quotient of the induced voltage *U* to the vertical velocity *ν*_*z*_ when the coil is moved vertically in the same magnetic field,

(2) $$-\frac{\partial \Phi }{\partial z}=\frac{U}{\nu_{z}}.$$

The essence of this insight is that a motor can be used as a generator, and the coupling constant between force and current is the same as the one between voltage and velocity. Quickly after Kibble’s proposal, the National Bureau of Standards (NBS, presently NIST) and the National Physical Laboratory (NPL) in the United Kingdom built balances to accommodate a moving mode.^4,5^

The derivative of the flux with respect to *z* is eliminated when combining both equations together leading to

(3) $$mg\nu_{z}=UI,$$

which describes an equivalence between virtual mechanical and electrical power. Since power is measured in its SI unit, the watt, the experiment and the apparatus came to be called a “watt balance.”

After the discovery of the quantum Hall effect in 1980 by von Klitzing,^7^ and two decades after the prediction of superconductive current tunneling between two metals in 1962 by Josephson,^8^ national metrology institutes started realizing the ohm and the volt with very high precision using these effects.

In the Josephson effect, a tunnel junction between two superconducting materials is radiated with microwave radiation with frequency *f*_*U*_. The potential difference between the two superconductors is given by $K_{\text{J}}^{-1}f_{U}$. Here, *K*_J_ denotes the Josephson constant given by $K_{\text{J}}=2e/h$, where *e* is the elementary charge and *h* the Planck constant. By using *n* junctions in series, a larger voltage $U=nK_{\text{J}}^{-1}f_{U}$ can be obtained.

The quantum Hall effect occurs in a sample that confines electronic current to two dimensions at sufficiently low temperatures. If such a sample is exposed to a high magnetic field, the quotient of the transverse voltage to the current flowing in the device, is quantized. This quantum Hall resistance is equal to an integer fraction of $R_{\text{K}}=h/e^{2}$. The constant *R*_K_ is named after von Klitzing and the quantum Hall resistance standards are realized in terms of the von Klitzing constant, $R=R_{\text{K}}/p$, where *p* is an integer.

The current *I* in Eq. (3) can be determined by measuring the voltage drop *U* across a resistor with resistance *R*. The equivalence between mechanical and electrical power can now be expressed as

(4) $$mg\nu_{z}=\frac{U^{2}}{R}.$$

Replacing *U* and *R* in Eq. (4) with the expressions from the Josephson effect (twice) and the quantum Hall effect yields

(5) $$mg\nu_{z}={\left\{\frac{pn^{2}}{4}f_{U}^{2}h\right\}}_{\text{SI}}\text{W}.$$

Conveniently, the elementary charge cancels out in the product $K_{\text{J}}^{2}R_{\text{K}}=4/h$. The quantity in {}_SI_ denotes the numerical value of power expressed in its SI unit, the watt, W.

However, there is a small subtlety that has to be considered. Both *K*_J_ and *R*_K_ depend on the elementary charge and the Planck constant. Our best estimate of the numerical values of these constants is subject to change depending on new research. In order to avoid changing the calibration of electrical equipment every time a new set of fundamental constants is recommended, the electrical metrology community decided to use a set of constants fixed at the 1990 values.^9^ These constants, denoted with the subscript 90, are *K*_J−90_ ≡ 483597.9 × 10^9^ Hz V^−1^ and *R*_K−90_ ≡ 25812.807 Ω. Both of these constants are used to realize the so called conventional units. The electrical power can now be written as

(6) $$\frac{U^{2}}{R}={\left\{\frac{pn^{2}}{4}f_{U}^{2}h_{90}\right\}}_{90}{\text{W}}_{90},$$

where the quantity in {}_90_ denotes the numerical value in conventional units.

Combining Eqs. (4)–(6) yields the expression

(7) $$\frac{mg\nu_{z}}{U^{2}/R}=\frac{{\left\{mg\nu_{z}\right\}}_{\text{SI}}\text{W}}{{\left\{U^{2}/R\right\}}_{90}{\text{W}}_{90}}=\frac{{\left\{\frac{pn^{2}}{4}f_{U}^{2}h\right\}}_{\text{SI}}\text{W}}{{\left\{\frac{pn^{2}}{4}f_{U}^{2}h_{90}\right\}}_{90}{\text{W}}_{90}},$$

that can be simplified into

(8) $$\frac{{\left\{h\right\}}_{\text{SI}}}{{\left\{h_{90}\right\}}_{90}}=\frac{{\left\{mg\nu_{z}\right\}}_{\text{SI}}}{{\left\{U^{2}/R\right\}}_{90}}.$$

This is the main watt balance equation. The Planck constant value *h* in SI units is determined from the ratio of the virtual mechanical power measured in SI units to the electrical power in conventional units. In order to reach relative uncertainties on the order of a few parts in 10^8^, the principle quantities in the experiment must be determined with relative uncertainties of a few parts in 10^9^. This is achieved by using, directly or indirectly, five standards: mass, voltage, resistance, time, and length.

**FIG. 1.**
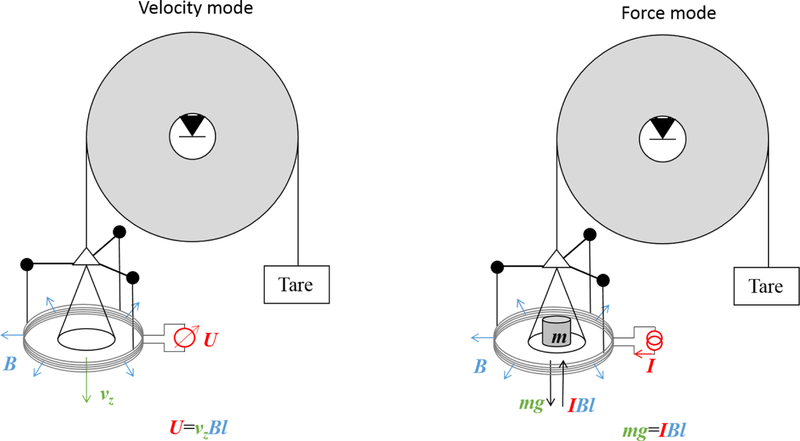
The principle of a watt balance using a pulley. In velocity mode (left), the coil moves vertical through the magnetic field. This process generates an induced voltage *U* which is proportional to the product of the vertical velocity *ν*_*z*_, the magnetic flux density *B*, and the wire length *l* of the coil. By measuring *U* and *ν*_*z*_, the flux integral *Bl* can be obtained by a simple division. In this figure, the drive force to move the pulley and hence the coil is a motor on the right side of the pulley (not shown in the picture). In the force mode (right), the electromagnetic force *I Bl* is generated by the coil carrying current *I* placed in the same magnetic field. The test mass *m* is positioned on the same side as the electromagnetic system in order to balance the torques of the electromagnetic and gravitational forces acting on the pulley independently from the lever arm. The current *I* in the coil is adjusted to maintain the position of the pulley to a nominal position.

In addition to the efforts at NIST^11–14^ and NPL,^15–17^ other national metrology institutes have taken the challenge to build watt balance experiments. These include the Swiss Federal Institute of Metrology (METAS),^18^ the Laboratoire National de Métrologie et d’Essais (LNE) of France,^19^ the Bureau International des Poids et Mesures (BIPM),^20^ the Measurement Standards Laboratory of New Zealand (MSL),^21^ and the Korea Research Institute of Standards and Science (KRISS).^22^ The National Research Council (NRC) of Canada purchased the NPL apparatus in 2011 and made improvements that lowered the overall uncertainty.^23^ The National Institute of Metrology (NIM) of China proposed a Joule balance, in which the derivative of the flux with respect to *z* is calibrated using a mutual inductance method instead of moving the coil in the magnetic flux.^24^ The principle of the watt balance experiment is presented in the simplified scheme of Fig. 1. Some common main elements in most watt balance experiments are the following:

1. *A coil in a magnet system*, where the magnet system is generally built using rare earth magnets with iron yokes but also can be an electromagnet with room temperature copper wires or superconducting wires as well as a hybrid. As recognized by Olsen,^6^ it is advantageous that (a) the magnetic flux density *B* has a spatial gradient inversely proportional to the coil radius. This eliminates systematic effects associated with geometry changes of the coil caused by resistive heating in the force mode, and (b) the radial magnetic flux density is uniform along the vertical motion, i.e., the force on the coil is independent of its vertical position. Both goals can be achieved by using two vertically separated electromagnet sources with a symmetry about the mid-plane. Most contemporary watt balance experiments have adopted this geometry, replacing the field coils as proposed originally with permanent magnets as suggested by the watt balance group at BIPM.^25^ A magnet system comprising permanent magnets with yokes presents two advantages: first, the magnetic flux is contained, and second, the coil placed in the air gap of the magnet system is shielded from the outside. In most recent papers, the derivative of the flux with respect to *z* is represented in its integral form. It is equal to the product of the magnetic flux density *B* and the wire length *l* of the coil, $\left(-\frac{\partial \Phi }{\partial z}=Bl\right)$. The product *Bl* will be referred in this paper as the flux integral.
2. *A moving mechanism* to translate the coil vertically in the magnetic flux. The deviations from a one dimensional motion should be minimized and characterized to estimate the biases that these parasitic motions introduce to the voltage and velocity measurement.
3. *A force comparator* sensitive to forces along the direction of the gravitational acceleration. It can be a pulley, a beam balance, or a commercial mass comparator. Two kinds of mechanical pivots are used: knife edge or flexure. A knife edge can provide a larger angle of rotation than a flexure with the same lever arm length. Hence, if a knife edge is used, the force comparator can play both roles that of the weighing and moving mechanism. Using one mechanism for moving and weighing not only simplifies the design but also has a significant technical advantage. The error motions in velocity mode correspond to virtual error motions in force mode. This symmetry significantly reduces the effect of these error motion on the final result, see Ref. 26 for details. The disadvantage of using a knife edge is that the large mechanical stress introduced in the velocity mode can cause time and position dependent mechanical forces in the force mode, known as mechanical hysteresis. The hysteresis is reduced by an erasing procedure where the pivot is exercised in a damped sinusoidal motion after each excursion. Flexures have small hysteresis if the motions are limited, but a separate mechanism to move the coil in the magnetic flux is required in the velocity mode, which makes the mechanical design more complex.
4. *An absolute gravimeter* to determine the local due to gravity. The transfer to the location of the test mass *m* is usually done with a relative gravimeter. To date, two different kinds of precise absolute gravimeters are employed in watt balance experiments: a classical interferometer measuring the acceleration of a free falling retroreflector^27^ or an atom interferometer.^28^
5. *Precise laser interferometer* to measure the vertical velocity of the coil in motion derived from absolute measurement of length and time interval. Typically, heterodyne and homodyne laser interferometers in a Michelson configuration are used, but a Fabry-Pérot interferometer can also be used as in METAS watt balance.
6. *Sensors and actuators* to monitor and control the remaining five degrees of freedom of the coil in the magnetic field.
7. *A programmable current source* to adjust the current in the coil in the force mode and maintain the equilibrium position of the force comparator. The equilibrium position can be measured by laser interferometry or other sensitive position sensors.
8. *A Josephson array* as the voltage standard. It can be a conventional hysteretic array or a programmable array. Most watt balances use a programmable Josephson voltage standard (PJVS), which offers the possibility to track the voltage of the coil in the velocity mode of the experiment during acceleration and deceleration to avoid overloading of the null detector. A PJVS requires a bias current source that should be electrically isolated to operate properly with the watt balance.
9. *Precision digital voltmeters* as null detectors to measure the deviation of the voltage drop across the resistor in the force mode and the induced voltage of the moving coil in the velocity mode from the voltage set by the Josephson array.
10. *A precision resistor* with low temperature and power coefficients. The resistance value is chosen based on the design choices of test mass, field strength, coil length, and setting range of the programmable voltage standard. Common values are 100 Ω or 200 Ω. The absolute value of the resistance is calibrated by comparison to a quantum Hall based resistance.
11. *A vacuum system* to house the watt balance in order to minimize corrections due to air buoyancy and index of refraction, and their related large standard uncertainties in air.
12. *Alignment tools and methods* to align the electromagnetic vector force ***F***, the coil velocity vector ***ν***, and the sensors that measure ***F*** and ***ν***, along *z* defined by ***g***.

A summary of the commonalities and highlight of the main differences between watt balance experiments, as well as the history and progress on accurate measurements of the Planck constant, were published in a recent paper.^10^ In Secs. II–IV, the recently built NIST watt balance apparatus is described, as well as the procedures to align the instrument, the measurement, and the data analysis. The Planck constant value measured from December 2015 to January 2016 is reported in Secs. V and VI.

## II. NIST’S FOURTH GENERATION WATT BALANCE APPARATUS

**FIG. 2.**
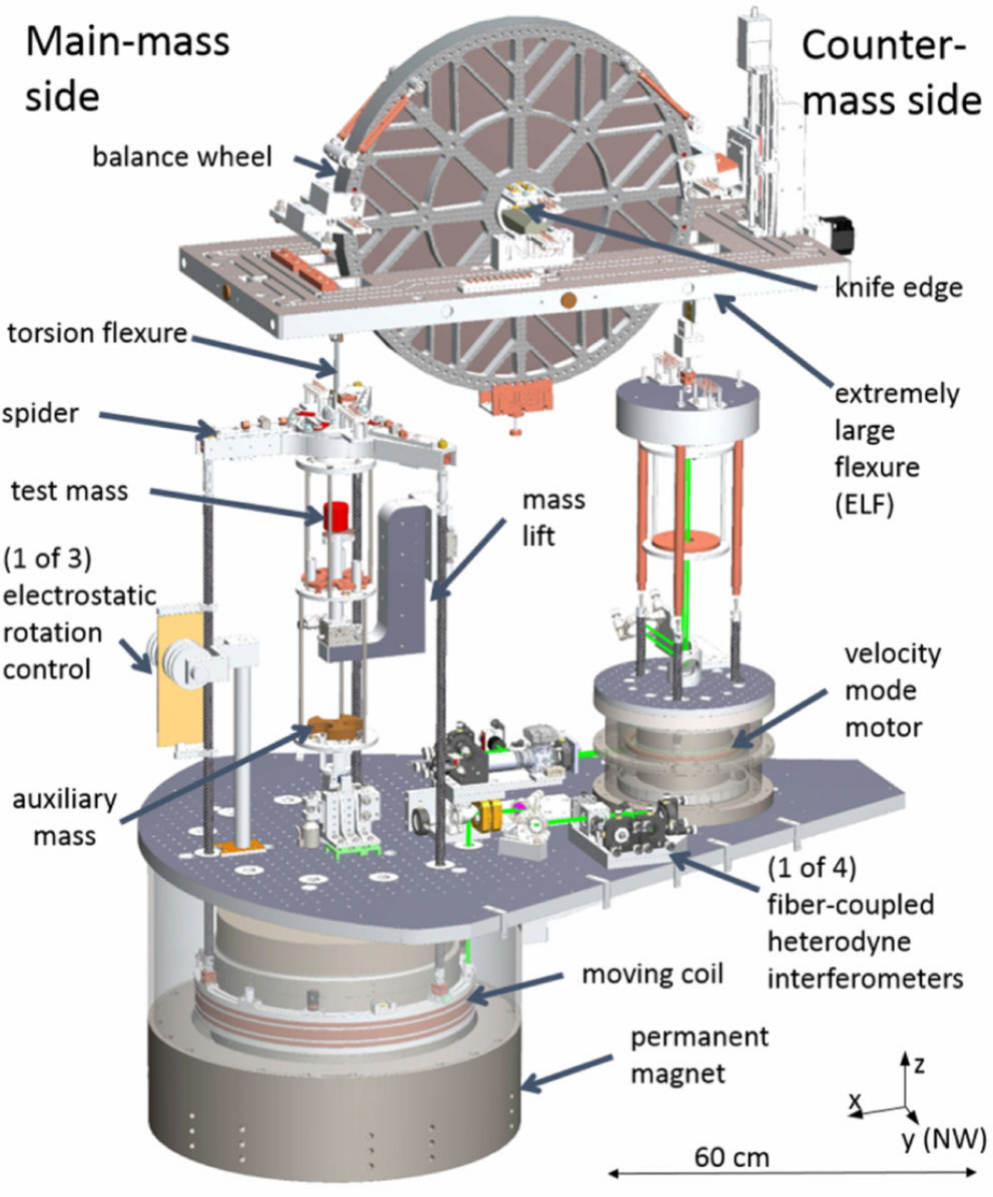
Three dimensional drawing of the NIST-4 watt balance. Everything to the left of the wheel’s center is referred to as the main-mass side. The opposing side is the counter-mass side. The large aluminum plate in the shape of a tear drop bolted to the top surface of the magnet is the base for mounting the optics, the building platform for the mass lift and counter-mass motor consisting of a coil in a small permanent magnet.

**FIG. 3.**
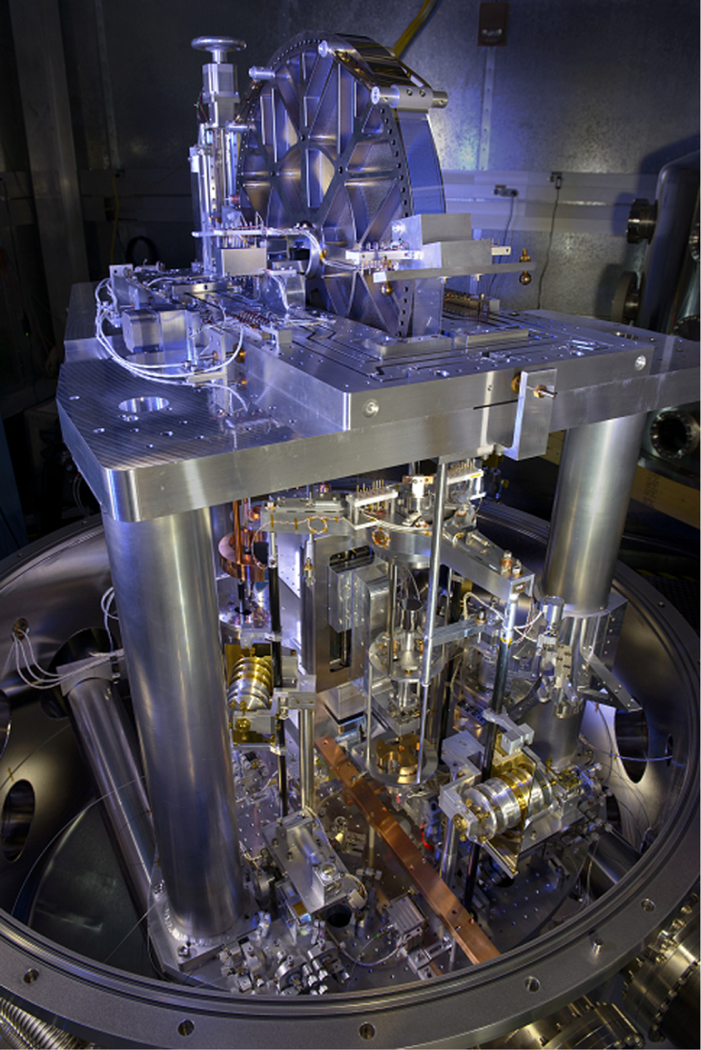
A close-up view of the main-mass side. The three-pointed aluminum star, named spider, is suspended from a torsion flexure. The electrical signals are brought to the main coil and six damping coils with eleven ultra-fine wires visible to the left and right of the torsion flexure. Three carbon fiber rods reach into the magnet below and connect to the alumina coil former. Gold coated plates attached to the carbon fiber rods are part of the electrostatic azimuthal rotation control. A standard mass is on the mass plunger raised above the mass pan. The bronze colored three-pointed star is the auxiliary mass. It is loaded on a dedicated mass pan which is rigidly connected to the main-mass pan.

The fourth generation watt balance apparatus, abbreviated NIST-4, shown in Figs. 2 and 3, is located inside a radiofrequency shielded laboratory, 12 m underground. For later reference, observe that the balance is oriented along a diagonal with respect to the compass direction of North as shown in the figure, with the main pivot axis falling along a line oriented from the north-west to south-east corners of the room. The room itself is temperature stabilized using an air jacket between the inner and outer exterior walls. Inside the room, the apparatus sits on the center of a concrete block that is 4 m long and 4 m wide and has a total mass of 67 metric tons. This block is isolated from the building’s foundation and, for additional vibration isolation, can be floated off the ground using eight air springs. The apparatus is housed inside a 1.6 m diameter and 2 m tall stainless steel vacuum chamber which is pumped to a vacuum pressure on the order of 0.1 mPa. The watt balance apparatus is supported at three points in a pseudo-kinematic fashion on top of two stainless steel, sand-filled, parallel tubes that run from south-west to north-east through the chamber. The tubes are structurally decoupled from the vacuum chamber via four flexible bellows. This minimizes the transmission of chamber vibrations to the apparatus and maintains the apparatus alignments from air to vacuum. We have verified the alignment of the top of the magnet in four different configurations: (1) with the vacuum chamber top lid open, (2) closed, (3) during the evacuation of the chamber and (4) in vacuum using an autocollimator with the beam reflecting from a mirror on the tear drop plate. Between these four cases, the angle between the top of the magnet and a horizontal plane did not change by more 30 μrad.

The main balance element is an aluminum wheel with a total mass of 18 kg and a diameter of 610 mm. The wheel was plated with electroless nickel and subsequently diamond turned. This resulted in a maximum deviation of 900 nm from roundness. The wheel pivots about a tungsten-carbide knife edge. A gimbal structure is suspended off each side of the wheel via multi-filament bands. Each band contains 60 filaments, each 130 μm wide and 317.5 mm long, photo etched from a 381 mm long sheet of 80 μm thick grade four titanium. Below the knife edge is a flat made from polished tungsten carbide and coated with diamond like carbon (DLC). The combination of knife edge and flat materials were selected based on previous NIST research results^35^ and a recent NPL paper.^36^ According to the latter findings, hysteresis performance of the knife edge pivot improves when the knife edge material or coating is softer than the flat. Consequently, the DLC coating was omitted from the knife, in contrast to previous approaches at NIST. The flat is mounted on an extremely large flexure(ELF),^33^ guiding the horizontal motion of the flat, that can be coarse positioned with adjustment screws in air. Two stepper motors provide fine positioning of the flat with a total range of about 0.5 mm in the *x* and *y* direction. The coil moves with the flat. With these two motors, the operator can adjust the position of the coil in the magnetic circuit while the apparatus is in vacuum.

The magnetic field is generated by an 800 kg permanent magnet system measuring 60 cm in diameter and 45 cm in height. Two Sm_2_Co_17_ disks oriented in repulsion generate, in a 30 mm wide air gap, a 0.55 T radial magnetic field guided by the iron yoke.^29,30^ While the magnetic flux density is mostly oriented along the radial direction, there is a small vertical component. This vertical field is caused by the finiteness of the gap (fringe fields at the end of the gap) and an asymmetry in the air-yoke boundary. An evaluation of the vertical component for the NIST-4 magnet can be found in Ref. 31. The ratio of the vertical component and the radial component is at most about 0.6% at *z* = ±50 mm, which would be even smaller (0.05%) in the NIST-4 measurement interval |*z*| < 25 mm. In the fine alignment of the apparatus, both the radial and vertical magnetic field will contribute. A detailed analysis of the influence of *B*_z_ in the alignment is described in Ref. 32. The mechanics of the watt balance is mounted directly to the top of the magnet system, so that the massive magnet system serves as a rigid structural foundation.

Suspended from the north-east side of the balance wheel is the main-mass assembly. A three-pointed aluminum spider connects to the end of the multi-filament band with an intermediate torsion flexure, a 60-strand bundle of 75 μm diameter platinum-tungsten wires. The mass pan has two levels, the main-mass pan and the auxiliary-mass pan. The two levels are rigidly connected by aluminum rods. The mass pan accepts the main mass from a plunger that reaches through a cut-out in the center of the main-mass pan. During velocity mode, the auxiliary-mass pan carries a 0.5 kg mass, named the auxiliary mass. Transitioning the instrument from velocity to force mode requires removal of the auxiliary mass from the pan system. This is accomplished with a lift mounted on a rotary stage below the auxiliary mass pan. Removing the auxiliary mass makes the main-mass side lighter by 0.5 kg. Hence, in order to maintain the wheel’s position, an electromagnetic force of 5 N must be generated by the main coil. Since the mass on the counter-mass side has not changed, the static load supported by the knife edge due to the transition from gravitational to electromagnetic force remains constant. This is a significant change from the previous NIST balance, implemented to improve the performance of the knife edge pivot.

Three carbon fiber rods attach the main coil to the spider leg via monolithic flexures at both ends having two orthogonal degrees of freedom each. A total of six flexures provide four different motions. The coil can tilt around the *x* or *y*-axis. For these two motions, the spider tilts with the coil because the three connecting rods are forcing the spider and the coil to be parallel all the time. The other two motions are a shear motion, i.e., the coil translates along the *x* or *y*-axis relative to the spider. The first two types of motions occur when a torque is applied to the coil. Horizontal forces on the coil cause shear motions. In reality, all four motions are present. The compliance of the system translates parasitic forces and torques of the energized coil into relatively large horizontal and angular displacements. From these recorded displacements, the parasitic forces and torques can be estimated. Both the mass pan and coil pivot each about independent monolithic flexures with two orthogonal degrees of freedom. Both flexures are nested to have all four axes of rotation coplanar and intersecting in a single virtual pivot. This monolithic flexure minimizes the coupling between the two suspended systems and aligns the electromagnetic and gravitational forces into a single point on the vertical axis.

**FIG. 4.**
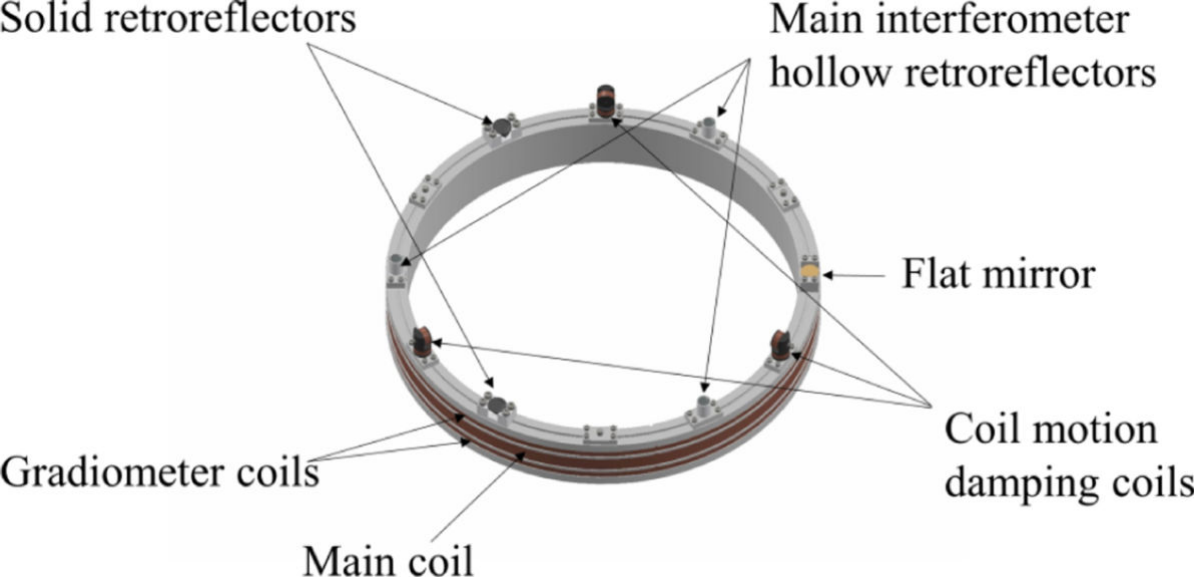
CAD model of the alumina former where the main and gradiometer coils are wound and nine motion devices are placed every 30°.

For the main coil, 945 turns were wound in the center of a monolithic coil former made of 99.5% alumina (Fig. 4). The coil has a mean radius of 0.217 m, a resistance of 112 Ω and a low frequency inductance of 6 H. Two additional gradiometer coils were wound above and below the main coil. The gradiometer coils are only used during the assembly of the magnet, where they provide a means to measure the vertical dependence of the radial magnetic flux density in the gap of the magnetic circuit. A shimming procedure has been developed that manipulates the reluctance of the yoke, and the variation of the radial flux density in the precision gap can be systematically adjusted to within less than 1 mT i.e.,

∆*B_r_*/*B_r_* < 2 × 10^−4^. See Ref. 30 for details on the shimming procedure. Prior to winding the coil, the coil former was painted with a static dissipative coating. To prevent closed-circuit eddy current loops, the coating was interrupted on three spots, 120° apart. Each of the three coated sections are grounded individually to eliminate static charge buildup.

**FIG. 5.**
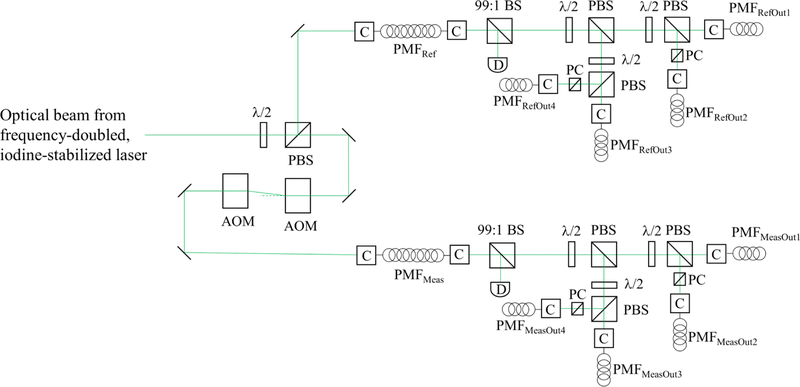
Conditioning of the light for the four interferometers outside the vacuum chamber. The light source for the heterodyne interferometers is a frequency-doubled, iodine-stabilized Nd:YAG laser at 532 nm which is one practical realization of the meter.^44^ The laser beam is split with a half-wave plate (*λ*/2) and a polarizing beam splitter (PBS) into a reference and measurement beam. The measurement beam passes through two acousto-optic modulators (AOM). The first AOM shifts the measurement beam frequency by 81 MHz and the second one shifts it by −79 MHz, yielding an optical frequency in the measurement beam to be 2 MHz above that of the reference beam. The reference and measurement beams are coupled each into polarizing maintaining fiber (PMF) with a coupler (C) and subsequently are split into four outputs with a *λ*/2 and a PBS then coupled into polarization maintaining fibers (PMF) to deliver the light to the three main interferometers and a fourth auxiliary interferometer inside the vacuum chamber. The small polarizing beam splitter cubes (PC) clean the state of polarization of the reflected beams. Two photodiodes (D) are used to monitor the alignment of the input fibers.

**FIG. 6.**
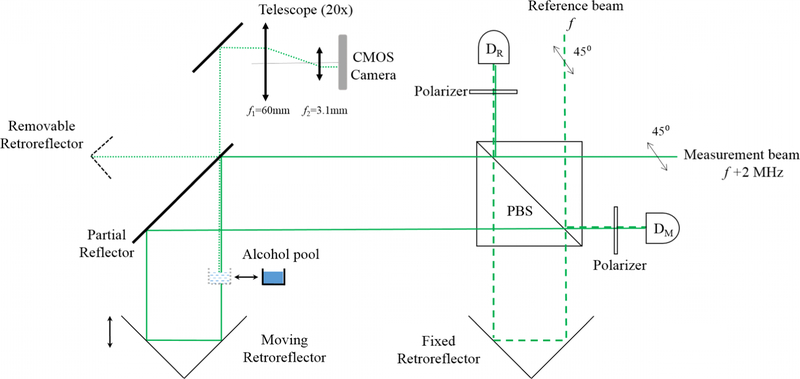
This schematic shows one of the three main fiber-coupled heterodyne interferometers as well as the tools to align to vertical the laser beam in air. The laser beams are transported from the outside to the inside of the vacuum chamber with polarizing maintaining fibers, having vacuum feed-through. The reference and the measurement beams are reshaped, linearly polarized at 45°, and then split equally by the polarizing beam splitter (PBS). Part of the reference beam is reflected directly to the measurement photodetector (D_M_), and the other part passes through the PBS and is reflected by a fixed retroreflector back on the reference photodetector (D_R_). Similarly, part of the measurement beam illuminates D_R_ and the other part is reflected from the moving retroreflector back on (D_M_). The reference signal on D_R_ is necessary to account for phase changes acquired in the fibers. To align the measurement laser beam vertically while in air, an alcohol pool giving a horizontal reference plane is inserted in the beam that goes down to the moving retroreflector on the coil. The laser beam reflected from the alcohol goes through the partial reflector, then the telescope to finally illuminates a CMOS camera. This beam is compared to a reference beam reflected from the apex of a temporarily inserted removable retroreflector. The partial reflector is adjusted to overlap both beams on the camera.

The six rigid-body motions of the main coil are monitored using optical techniques. A set of three hollow retroreflectors is installed around the coil circumference 120° apart and form the measurement arms for the fiber-coupled heterodyne interferometry system as depicted in Figs. 5 and 6. This system is dedicated to measuring the absolute translation of the optical center of the coil along an axis aligned to gravity. Another set of three optical elements is similarly installed around the coil circumference at 120° intervals, but offset from the first by 60°. This set consists of two solid retroreflectors and a flat mirror. The two solid retroreflectors are used in an optical beam translation scheme to monitor horizontal displacements and angular motion of the coil about the *z*-axis. The single flat mirror is used with an optical lever scheme to measure angular motions about the *x* and *y* axes. The reference light beams in both the beam translation and optical lever setups are amplitude modulated to facilitate lock in detection of the reflected light on position sensitive photo-detectors located on the top of the magnet.

Motion of the coil is weakly constrained along all but the *z*-axis, so servo control loops are required around the other five degrees of freedom during various operations to damp unwanted oscillations at various points during a measurement. The position signals from the previous paragraph are the control inputs. To control the rotations about the main-translation axis *z*, three glass plates coated with gold are attached vertically to each carbon fiber rod. These plates are sandwiched between sets of high-voltage electrodes fixed to the magnet, as shown in Figs. 2 and 3, to form a bi-directional electrostatic actuator. To actuate the remaining degrees of freedom, three sets of mini coils are mounted on the coil itself, between the optical elements, 120° apart to actively damp the horizontal and angular motions about *x* and *y*. Each mini coil set contains one vertically and one horizontally oriented coil. The damping coils are wound on carbon-filled, static dissipative polyether ether ketone (PEEK) coil formers, with a diameter of 18 mm. They are driven individually using single-ended, bipolar, 16-bit digital-to-analog converters. However, two coils in one set share a ground to minimize the wire counts. Hence, only three wires for each set are required. The output channels are calculated by software to excite pure horizontal or angular motion about *x* and *y* axes.

A variety of electrical connections to the instrument are required, the most difficult being those that must reach the main coil and damping coils, since these wiring connections can impart parasitic rotations of the coil about *x* and *y* axes and rotation of the balance wheel. Eleven wires, nine for the damping coils and two for the main coil, are routed inside the carbon fiber tubes that connect the coil to the spider. The distance from the spider to the wheel, about 30 cm, is bridged using finer wires. The wires terminate on blocks mounted on the balance wheel such that the wires are coplanar with the bands running off the wheel. The electrical signals are carried with thicker wire along the radius vector of the wheel to its center. Fine wires are used again to bridge from the moving wheel to the fixed wheel support. Here the eleven wires are arranged such that they are along a line extending from the knife edge ridge. The wire routing was chosen to minimize torques produced by the wires about the knife-edge and to keep this torque independent of wheel angle.

The motors that operate the mass lift dissipate heat during the weighing cycle. A thick copper bar is installed on top of the magnet as a thermal link to transport unwanted heat to the outside.

Suspended from the south-west side of the wheel is the counter-mass assembly. This structure serves both as a tare weight and as the velocity mode driving motor. The motor consists of a small coil wound on a G10 fiberglass former and a radial magnetic field generated by two neodymium disks bound by iron yoke, a smaller but similar design to the main magnet. A large aluminum cylinder forms the top part of the counter-mass assembly. A corner cube is mounted facing down on the lower side of the cylinder. This corner cube terminates the measurement arm of an interferometer whose readout is used as an input for the balance feedback.

For all four heterodyne interferometers, three on the main side and one on the counter-mass side, time interval analyzers measure the zero-crossings of the reference and the measurement signals. Along with the time interval measurements, continuous event counts of the reference and the measurement signals is maintained on all channels in order to keep track of the coil position during the measurement.

A custom built programmable current source^34^ operates in a feedback loop to generate the necessary current, *I*, flowing through the coil to maintain the position of the balance in the force mode. A similar current source is used for the counter-mass motor in the velocity mode. Both current sources are floating, since they are battery powered and connect to the control computer via and optical fiber link. They feature low noise on the order of 100 $\text{pA}/\sqrt{\text{Hz}}$ at 1 Hz and a relative short-term drift less than 0.1 (nA/mA)/h.

**FIG. 7.**
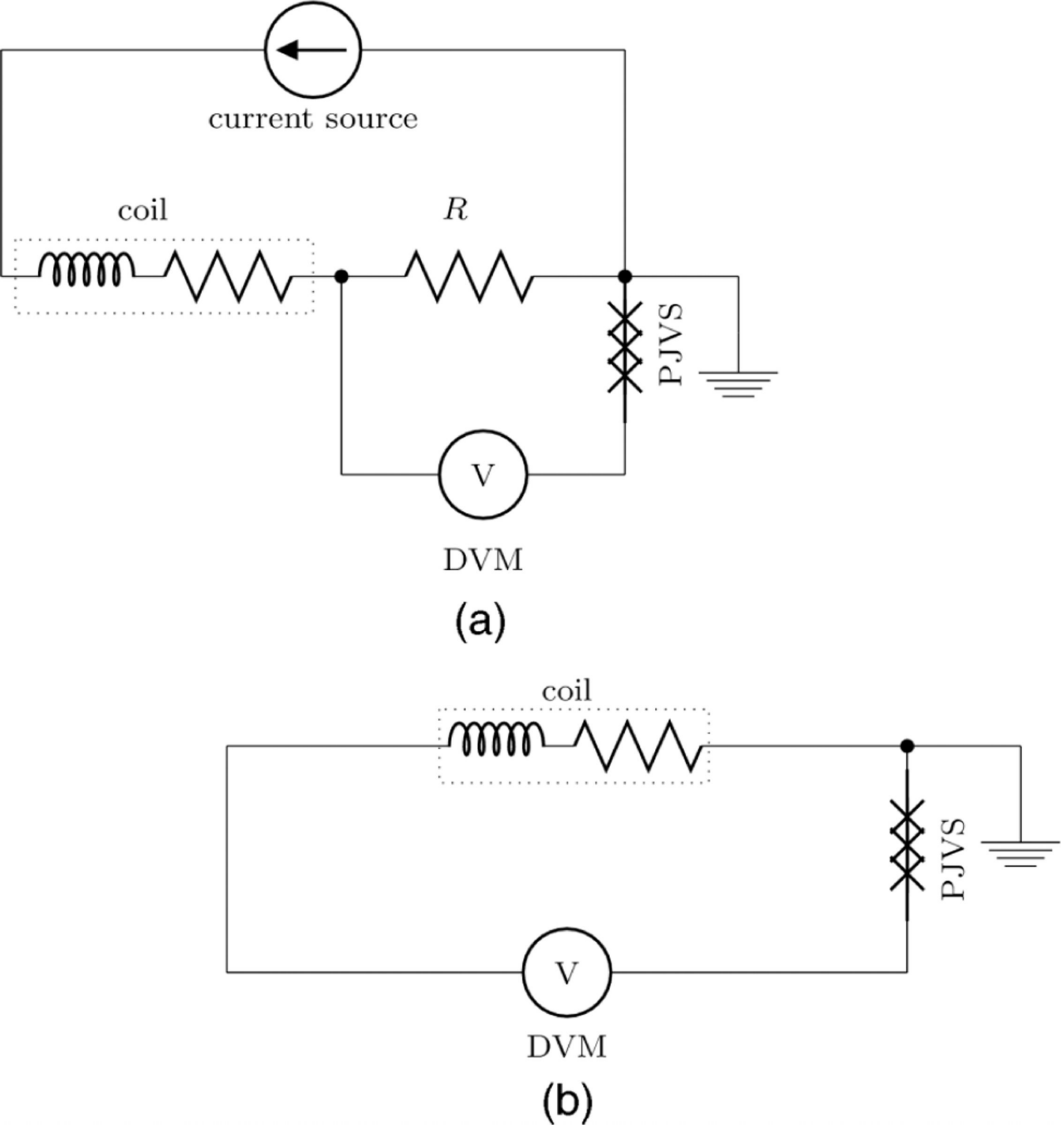
The electrical circuit in the force mode and velocity mode.

To reconfigure the electrical circuit between force and velocity mode shown in Fig. 7, a switchbox, featuring low thermal electromotive force (EMF) latching relays are used. The relays are only energized during mode switching and the circuit is completely turned off during the measurement.

A 2.5 V primary voltage system based on a NIST-fabricated programmable array of Josephson junctions and developed electronics^40,41^ is used as the programmable Josephson voltage standard (PJVS).^42^ The leakage resistance to ground of the non-battery powered bias electronics was improved with an air gap isolation transformer.

A ten megahertz clock signal derived from a Cesium clock frequency standard is used for the Programmable Josephson voltage standard counter. To avoid ground loops, the time interval analyzer and the absolute gravimeter temporarily use a Global Positioning System (GPS) disciplined oscillator.^43^ Both clock signals are routinely compared.

## III. ALIGNMENT PROCEDURES

**FIG. 8.**
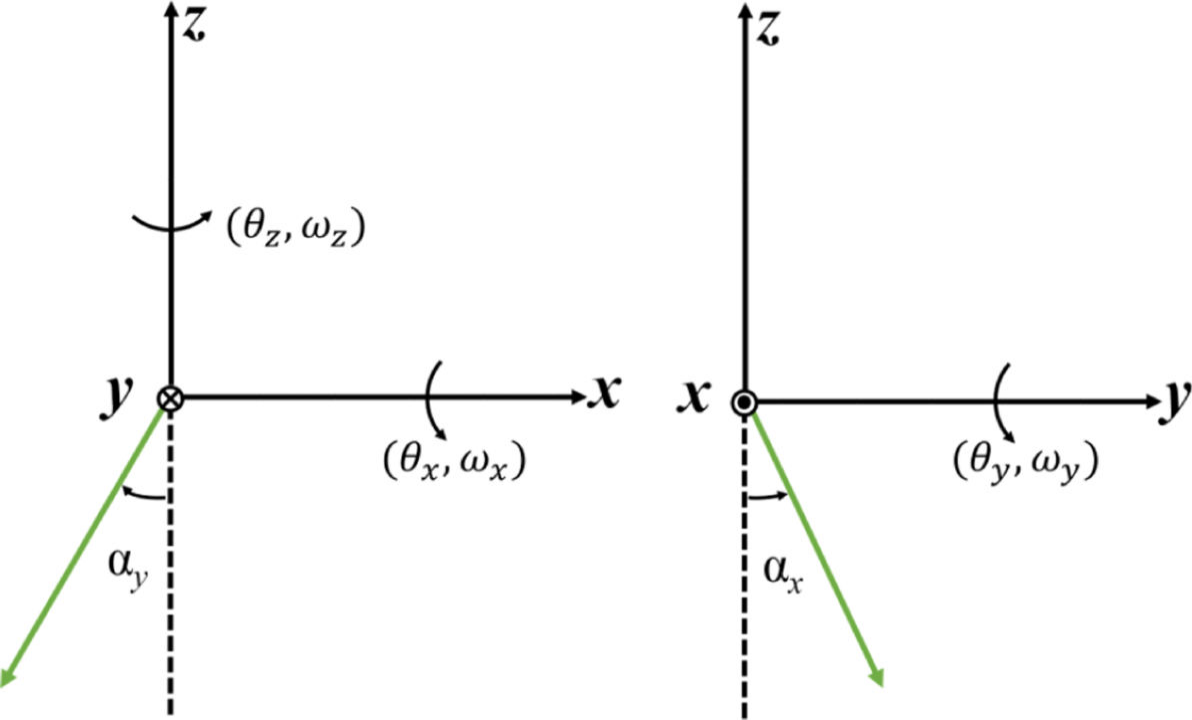
Angular displacements and velocities of the coil, and the laser beam misalignments from vertical in the Cartesian coordinate system. For instance, the angular rotation and velocity of the coil about *z*-axis are denoted *θ*_*z*_ and *ω*_*z*_. The misalignment of the laser beam from vertical about the *x*-axis is represented by *α*_*x*_. The direction of the angular rotation is according to the right-hand rule.

Extensive alignment procedures have been described in many articles.^37–39^ Here, we present a brief description of our alignment methods and uncertainties. The velocity vector of the coil ***ν***(*ν*_*x*_,*ν*_*y*_,*ν*_*z*_,*ω*_*x*_,*ω*_*y*_,*ω*_*z*_) and the directions of the laser beams that measure the coil velocities have to be aligned along the vertical direction *z* defined by ***g***. The angular displacements and velocities of the coil, and the misalignment from vertical of the laser beams are depicted in Fig. 8.

We start by aligning the laser beams of the three heterodyne interferometers to vertical as shown in Fig. 6. These interferometers measure the vertical velocity *ν*_*z*_, and the angular velocities around *x* and *y*,*ω*_*x*_ and *ω*_*y*_. A misalignment *α*_*x*_ and *α*_*y*_ from vertical of the laser beams will cause a cosine error approximated for small angles as quadratic error. Due to the angular misalignment of the laser beams from vertical, additional errors occur caused by the motions of the hollow retroreflector of the interferometer measurement arm in presence of horizontal velocities *ν*_*x*_ and *ν*_*y*_, and angular velocity *ω*_*z*_ of the coil. The alignment of the laser beams to vertical is performed with the system vented and the vacuum dome removed. Each of the laser beams directed toward the moving hollow retroreflectors attached to the coil is intercepted by an alcohol pool. A retroreflector, that is not part of the interferometer and that is removed during normal operation, intercepts the laser beam transmitted by the partial reflector. Both reflected laser beams from the apex of the removable retroreflector and the alcohol pool travel through a telescope with an angular magnification of 20 to finally shine on a complementary metal-oxide semiconductor (CMOS) camera with a pixel resolution of 5.2 μm. Note that we gain a factor of 2 in the angular magnification due to the fact that the angle between the reflected laser beam from the hollow reflector and the one from the alcohol pool is twice the angular misalignment of the laser beam. Hence the effective angular magnification is 40.With this scheme, we can align the beams to vertical with a standard uncertainty better than 40 μrad. The uncertainty in the angle determination between the laser beams and vertical is mainly due to the motion of the alcohol pool reflection spot on the camera.

In a second step, we align to vertical the laser beams that are directed toward the solid retroreflectors attached to the coil. The positions of the reflected laser beams are monitored with position sensitive photodetectors. The horizontal velocities of the coil *ν*_*x*_, *ν*_*y*_ and the angular velocity *ω*_z_ are determined by calculating the numerical derivatives of the position measurement with respect to time.

The horizontal velocity *ν*_*x*_ is minimized by adjusting the rotation center defined by the knife edge with respect to the geometric center of the wheel. The velocity *ν*_*y*_ is minimized by adjusting the angular rotation of the wheel around the *x* axis. This is accomplished by shimming the flat supporting the knife edge. The fine wires connecting the main coil and damping coils to the fixed part of the apparatus are adjusted and/or annealed to minimize their effect on the angular velocities *ω*_*x*_ and *ω*_*y*_ of the coil during a velocity sweep. The three gold plates, part of the electrostatic azimuthal rotation control, are aligned to vertical so as to provide only tangential forces. The rotation control reduces the angular velocity *ω*_*z*_.

The vertical velocity of the coil is determined at the optical center, defined by the location of the three hollow retroreflectors. The optical center must be on the vertical line passing through the mass center of the coil, in order to minimize the Abbe offset errors when the coil has angular motions about *x* and *y*. The Abbe offsets *d*_*x*_ and *d*_*y*_ are determined by exciting the coil’s rotation about *y* and *x* respectively. Then, the vertical velocity *ν*_*z*_ is obtained by a linear combination of the three interferometers. The weight of each interferometer is chosen such that the effective optical center is on the vertical line passing through the mass center. Hence, the Abbe offset errors in the velocity determination are zero and we state only the relative standard uncertainty in Table I.

The scalar voltage, induced during the velocity mode, is caused in principle, by the six different velocity components of the coil. It is given by

(9) $$\begin{array}{l} U=-(\frac{\partial \Phi }{\partial z}\nu_{z}+\frac{\partial \Phi }{\partial x}\nu_{x}+\frac{\partial \Phi }{\partial y}\nu_{y}\\ +\frac{\partial \Phi }{\partial \theta_{x}}\omega_{x}+\frac{\partial \Phi }{\partial \theta_{y}}\omega_{y}+\frac{\partial \Phi }{\partial \theta_{z}}\omega_{z}). \end{array}$$

Except for the first term in the parenthesis, all terms are considered parasitic. The partial derivatives of the magnetic flux Φ with respect to *x*, *y*, θ_x_, and θ_y_, are estimated in the force mode as follows: the vector force ***F*** and torque ***τ*** on the main coil energized with current *I* are given by

(10) $$F=F_{x}\hat{x}+F_{y}\hat{y}+F_{z}\hat{z}=-I\left(\frac{\partial \Phi }{\partial x}\hat{x}+\frac{\partial \Phi }{\partial y}\hat{y}+\frac{\partial \Phi }{\partial z}\hat{z}\right),$$

(11) $$\tau =\tau_{x}\hat{x}+\tau_{y}\hat{y}+\tau_{z}\hat{z}=-I\left(\frac{\partial \Phi }{\partial \theta_{x}}\hat{x}+\frac{\partial \Phi }{\partial \theta_{y}}\hat{y}+\frac{\partial \Phi }{\partial \theta_{z}}\hat{z}\right).$$

Replacing the partial derivatives in Eq. (9) with the expressions from Eqs. (10) and (11) yields

(12) $$UI=F_{z}\nu_{z}\left(1+\frac{F_{x}}{F_{z}}\frac{\nu_{x}}{\nu_{z}}+\frac{F_{y}}{F_{z}}\frac{\nu_{y}}{\nu_{z}}+\frac{\tau_{x}}{F_{z}}\frac{\omega_{x}}{\nu_{z}}+\frac{\tau_{y}}{F_{z}}\frac{\omega_{y}}{\nu_{z}}+\frac{\tau_{z}}{F_{z}}\frac{\omega_{z}}{\nu_{z}}\right).$$

**FIG. 9.**
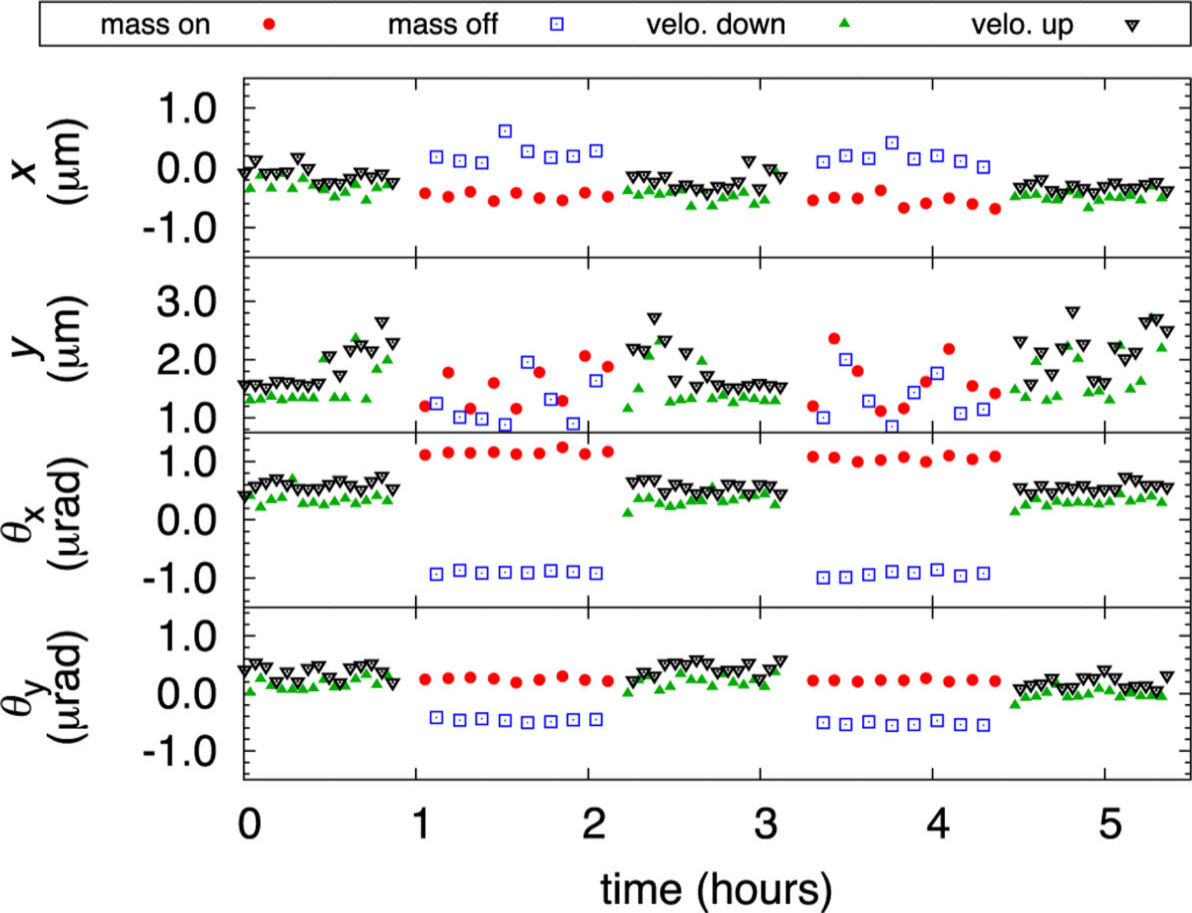
Position of the coil along four parasitic coordinates *x*, *y*, *θ*_*x*_, and *θ*_*y*_ in the velocity and force mode. The red and blue data points show the coil position in force mode during mass on and mass off, respectively. The green and black data points correspond to the position of the coil at *z* =0 during the velocity sweep.

The parasitic torques *τ*_*x*_ and *τ*_*y*_ are present due to the radial component of the magnetic flux density *B*_*r*_. These torques are non-zero if the electrical center of the energized coil is horizontally displaced from the magnetic center of the magnet system or if the electrical center is not on the vertical line passing through the coil’s mass center.The parasitic forces *F*_*x*_ and *F*_*y*_ are the consequences of the vertical component of the magnetic flux density *B*_*z*_ when the energized coil is tilted in the magnetic field. To observe these parasitic angular and horizontal motions, we alternate current between ±10 mA in the main coil. Then we move the coil with respect to the magnet system and add small masses on top of the spider to adjust the coil tilt to coincide the magnetic center and the electrical center of the coil on the vertical line passing through the mass center. The parasitic motions in a typical 5.5 h measurement are shown in Fig. 9. The typical error terms in the voltage measurement resulting from the parasitic motions of the coil are given in Table II.

## IV. MEASUREMENT PROCEDURE AND DATA ANALYSIS

Once the alignment steps are completed in air, the magnet is heated 30 min before and during the evacuation of the chamber until the temperature of the magnet reaches the operating temperature, about 23.3°C. This minimizes the temporal drift of the magnetic flux density *B* due to the rapid cooling of the magnet during the evacuation of the vacuum chamber. The vacuum pressure reaches 10^−4^ Pa after 24 h of pumping. Even though the magnetic flux density is still drifting by about 2.5 parts in 10^6^ h^−1^, we begin collecting data after 2 h of pumping. At this time, the pressure in the chamber is below 0.1 Pa and below the corona region in order to turn on the high voltage for the electrostatic rotation control.

**FIG. 10.**
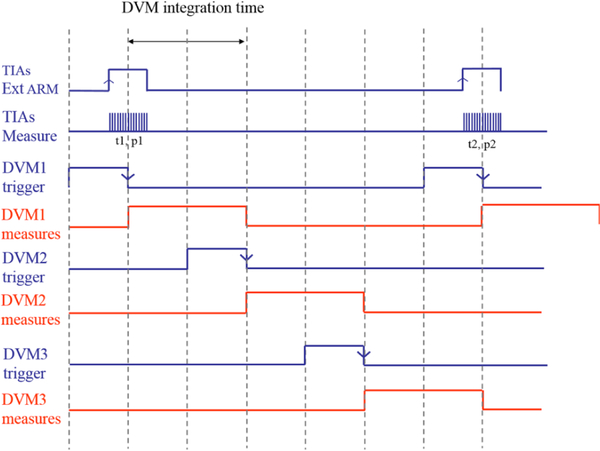
Triggering scheme in both force and velocity modes. For continuous voltage recording, three digital voltmeters (DVM) are triggered successively. The voltage is obtained by averaging the three DVM readings. To minimize the effect of frequency leakage in the arm of the interferometer, each of the three time interval analyzers records multiple readings per trigger, timed to equal one fringing period. The *λ*/2 optical fringe-crossing frequency is about 3.76 kHz, achieved by moving the coil at a velocity of 1 mm/s. Each velocity is calculated from two consecutive times and positions, at the center of the second DVM measurement.

In the velocity mode, the motions of the main coil are actively damped at the beginning of the velocity sweep to minimize the horizontal and angular motions about *x* and *y*. The coil is accelerated by 0.5 mm s^−2^ to the target velocity of 1 mm s^−1^ for the velocity sweep. At this point, the damping coils are opened by relays. The coil is swept in the magnetic field by 80 mm corresponding to a wheel rotation of about ±7.5°. Three digital voltmeters, triggered successively with auto-zero function as depicted in Fig. 10, measure the induced voltage across the main coil reduced by the voltage produced by the PJVS. The voltage, position, and velocity are integrated over multiple periods of both the power line cycles and the ground vibration.

In the force mode, the auxiliary mass is removed from the mass pan. This creates a force imbalance equal to half the weight of the standard mass. The current flowing in the main coil to keep the balance at a fixed position is determined by comparing the voltage drop across the standard resistor and the PJVS. The PJVS voltage is adjusted to have residual voltage difference of few 03BCV measured with three voltmeters using the same triggering scheme as in velocity mode. After the auxiliary mass is removed, the standard mass is placed on and off the mass pan 17 times. A mass exchange causes a small balance excursion, typically 45 μm. This excursion could alter the static restoring torque produced by the knife edge. The knife edge is not an ideal, frictionless pivot. Instead, it provides a small amount of fixed restoring torque. As long as this restoring torque is constant in time, the experiment does not suffer a bias. A small motion in the knife edge occurs with the balance excursion and has the potential to alter the restoring torque. Since the excursions are slightly different for the two different motions, by adding and removing the mass from the mass pan, a systematic bias can be created. To avoid such a bias, an erasing procedure is executed after each mass exchange. In the erasing procedure, the coil is moved along a trajectory given by a damped sinusoidal motion with an amplitude of 380 μm, and a decay time of 20 s to reduce the knife edge hysteresis.

A run is composed of multiple sets of flux integral measurements in the force and the velocity modes. Each set in the force mode consist of 17 current measurements, 9 with mass on and 8 with mass off. Each force mode set is bracketed by two velocity mode sets. Each set in the velocity mode comprises 30 coil sweeps. The direction of the coil velocity alternates between two successive measurements with the coil traveling down in the first measurement. The weighing position, chosen at the very beginning of the measurement, is derived from an initial calibration of the flux integral where the profile is flat. Then the counter-mass interferometer controlling the position and the velocity is set to zero at the weighing position, as well as the three main interferometers measuring the vertical and angular velocities of the coil.

**FIG. 11.**
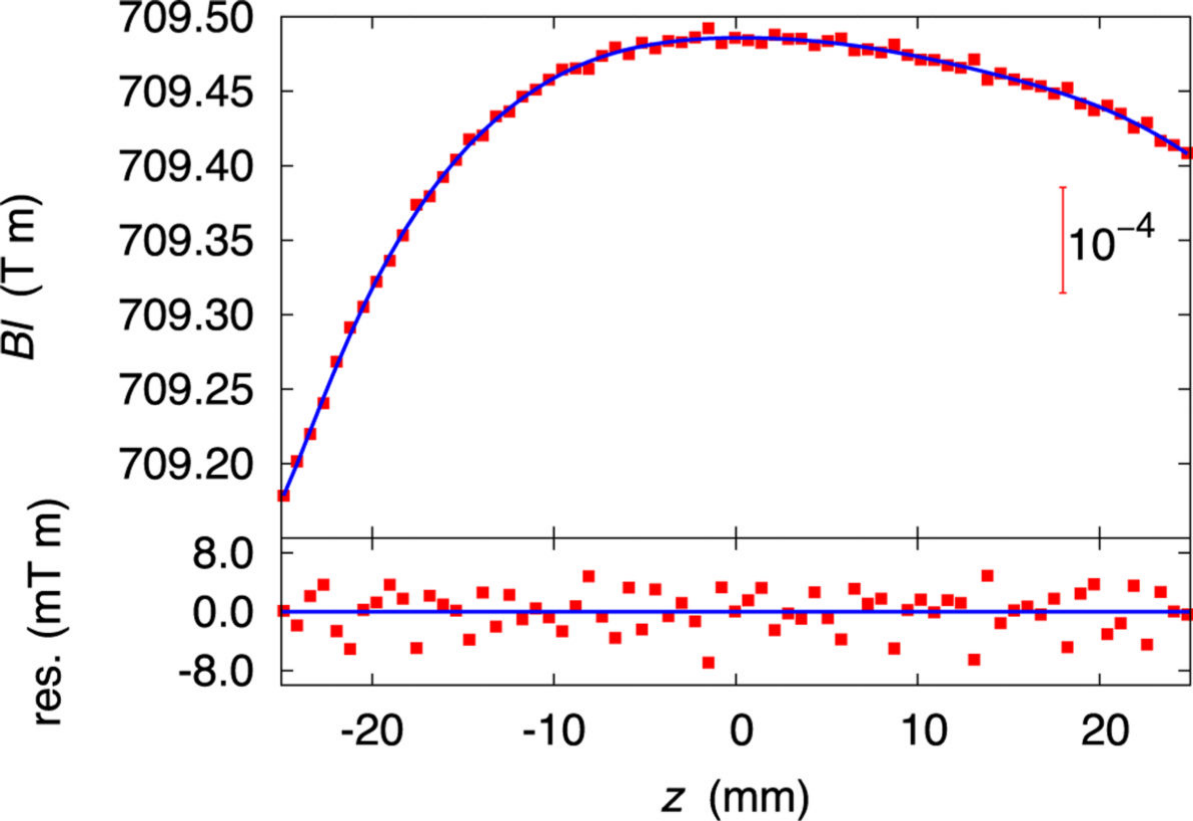
The upper graph shows the measured flux integral as function of the vertical position *z* in velocity mode. The asymmetry about *z* =0 is due to either the difference in field strength or dimension of the Sm_2_Co_17_ magnet rings. The lower graph shows the difference between the measured flux integral and the 11th order orthogonal polynomial fit.

For the velocity mode, the velocities are obtained by calculating a numerical derivative of the position. The voltage setting of the PJVS is added to the voltmeter reading and this number is divided by the velocity of the optical center. The result is the flux integral *Bl* as a function of the coil position *z* (Fig. 11). In one velocity sweep, 93 data pairs *z*,*Bl* are obtained. For each sweep, the coil position is transformed into the interval [−1 : 1] and a linear regression in terms of Chebyshev polynomials of the first kind is calculated. From all velocity sets in one run, a master profile is calculated by averaging the coefficients of all sweeps. The zero-order coefficient provides only a constant offset and is ignored for the master profile. The zero-order coefficient which is the result of each velocity sweep is then obtained by fixing all higher-order coefficients of the Chebyshev polynomials in the linear regression. Averaging the profiles to a master profile improves the statistical scatter of the velocity data by a factor of two. A comparison of calculations of the final result with and without the master profile proved that using the master profile does not introduce a bias to the final result. The result of two successive velocity sweeps (in one the coil moves up, in the other the coil moves down) are averaged into one number. This procedure cancels the thermal voltage that is generated mostly in the wires connecting the PJVS chip to room temperature. The thermal voltage is typically between 50 nV and 300 nV. The measured values of the flux integral in the velocity mode are also corrected for the weighing position due to stretching in the suspension system. At the end, each velocity mode set produces 29 (correlated) flux integral determination *Bl_V_*.

In the force mode, 78 voltage readings across the resistor *R* (nominally 100 Ω) are taken in 58 s. To this measurement the voltage produced by the PJVS is added. These readings are averaged to a single voltage reading. In addition to the average voltage reading, the average position of the coil (*x*, *y*, *z*, *θ*_*x*_, *θ*_*y*_, and *θ*_*z*_) is stored. The voltage is calculated into a current using *I* = *U*/*R*. The currents are obtained for the two states in force mode *I*_on_ and *I*_off_. Three adjacent force mode measurements, e.g., mass on, mass off, and mass on, are combined for each single determination of the flux integral in the force set. Each force mode set produces 15 flux integral determination *Bl*_*F*_.

Finally, we group together all flux integral measurements during one force mode and the flux integral measurements in the velocity mode measurements before and after this force mode measurement (29 *Bl*_*V*_, 15 *Bl*_*F*_, 29 *Bl*_*V*_). Hence, one triplet group consists of 58 *Bl*_*V*_ and 15 *Bl*_*F*_ measurements. To this data, a linear regression to the function $Bl(t)=\lambda_{1}+\lambda_{2}t+\lambda_{3}t^{2}+\lambda_{4}s(t)$, where *λ*_i_ are the regression coefficients and *s*(*t*) = 0 during velocity mode and *s*(*t*) = 1 during force mode. The parameter *λ*_4_ is the calculated difference between the numerical quantities, denoted by {}, of {*Bl*_F_} and {*Bl*_V_} and corresponds to the relative difference of *h* to *h*_90_, as shown below

(13) $$\frac{\left\{Bl_{F}\right\}}{\left\{Bl_{V}\right\}}=\frac{h}{h_{90}}=1+\frac{h-h_{90}}{h_{90}},$$

(14) $$\frac{\left\{Bl_{F}\right\}}{\left\{Bl_{V}\right\}}=\frac{\left\{Bl_{V}+\lambda_{4}\right\}}{\left\{Bl_{V}\right\}}=1+\frac{\left\{\lambda_{4}\right\}}{\left\{Bl_{V}\right\}}.$$

Hence,

(15) $$\frac{h-h_{90}}{h_{90}}=\frac{\left\{\lambda_{4}\right\}}{\left\{Bl_{V}\right\}}.$$

The result of one regression, λ_4_, is one data point in Fig. 14. The relative statistical uncertainty of *h* of one data point, inferred from the standard deviation of the whole data set, is 28 × 10^−9^. The measurement time required for one data point is about 3 h.

## V. EXPERIMENTAL RESULTS

The measurement of the Planck constant conducted from mid-December 2015 to early January 2016 was bracketed by both calibrations of the mass and resistor. The deviations of the laser beams from vertical were checked at the beginning and the end of the measurement. The determination of ***g*** was conducted in parallel to the measurement campaign.

**FIG. 12.**
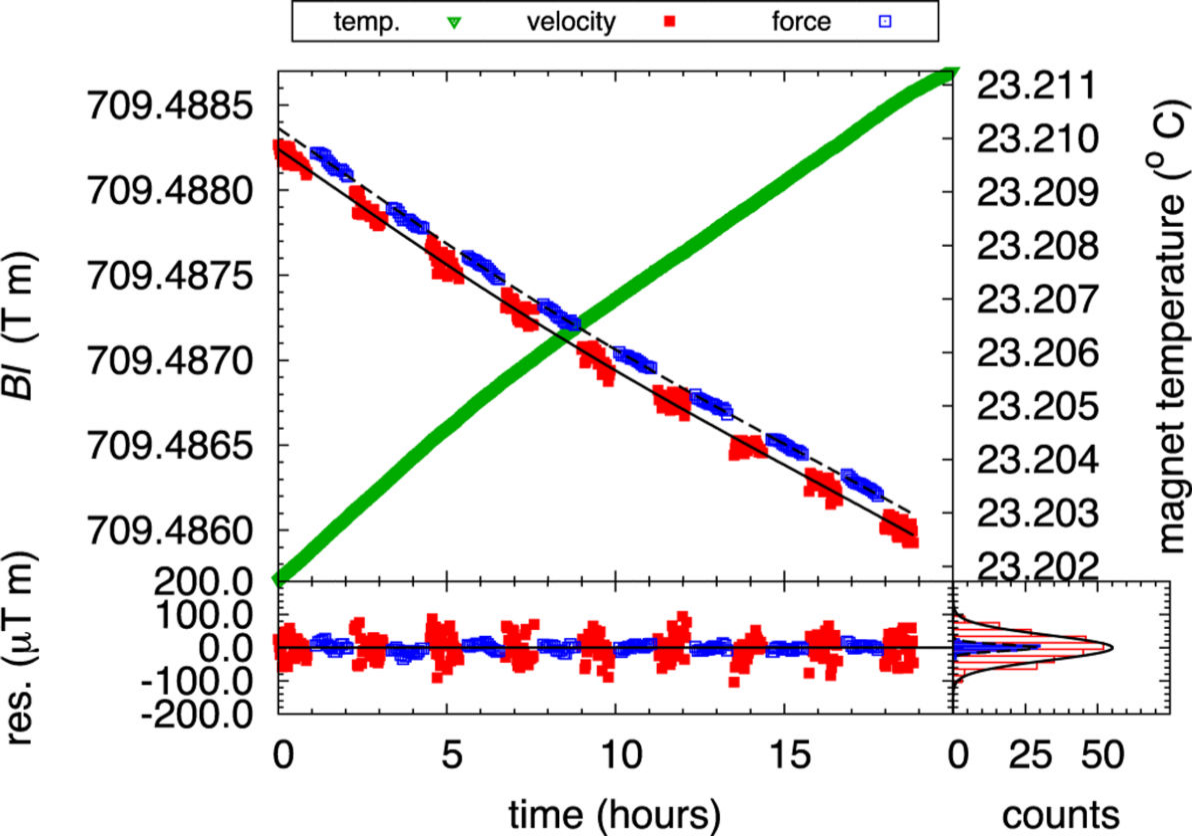
Measurements of the flux integral. The top graph shows in blue the measurement of the flux integral calculated from the force mode and in red the one calculated during the velocity mode. The temporal drift is caused by temperature change of the Sm_2_Co_17_ magnet. One can estimate the temperature coefficient of the magnetic flux density in the radial direction from the magnet temperature in green to be $\frac{\Delta B_{r}/B_{r}}{\Delta T}=-352\times 10^{-6}{\text{K}}^{-1}$. The lower left graph shows the difference between the measured flux integral calibrated in both velocity and force mode, and an exponential fit. It shows that the relative standard deviation of the flux integral in velocity mode is about 37.4×10^−9^ T m/T m and 9.8×10^−9^ T m/T m in force mode.

The typical noise of the flux integral in the velocity mode has a standard deviation of about 37.4 × 10^−9^ T m/T m, four times larger than the force mode (Fig. 12). Initially, we thought it was due to ground vibration, but even after reducing the vibration noise by a factor of 8, we did not observe a reduction in noise of the flux integral, additionally proving that the synchronization between voltage and velocity measurement is good. This additional noise in the dynamic operation of the balance is still under investigation, it is very likely caused by small variations in the trajectory for each sweep.

The standard mass used in this measurement is a 1 kg Pt-Ir prototype, K85.

Two different resistors with a nominal value of 100 Ω were used in this campaign of measurements. The first one, F005, chosen for its low power and temperature coefficients, is kept in a (25 ± 0.001) °C air bath. The second one, 1207, has more calibration history and was kept in an oil bath at (25 ± 0.001) °C. Both were calibrated against a quantum Hall effect (QHE) system without moving the resistors or the baths. The stability of the resistors was monitored against a 100 Ω bank, traceable to the QHE system.

**FIG. 13.**
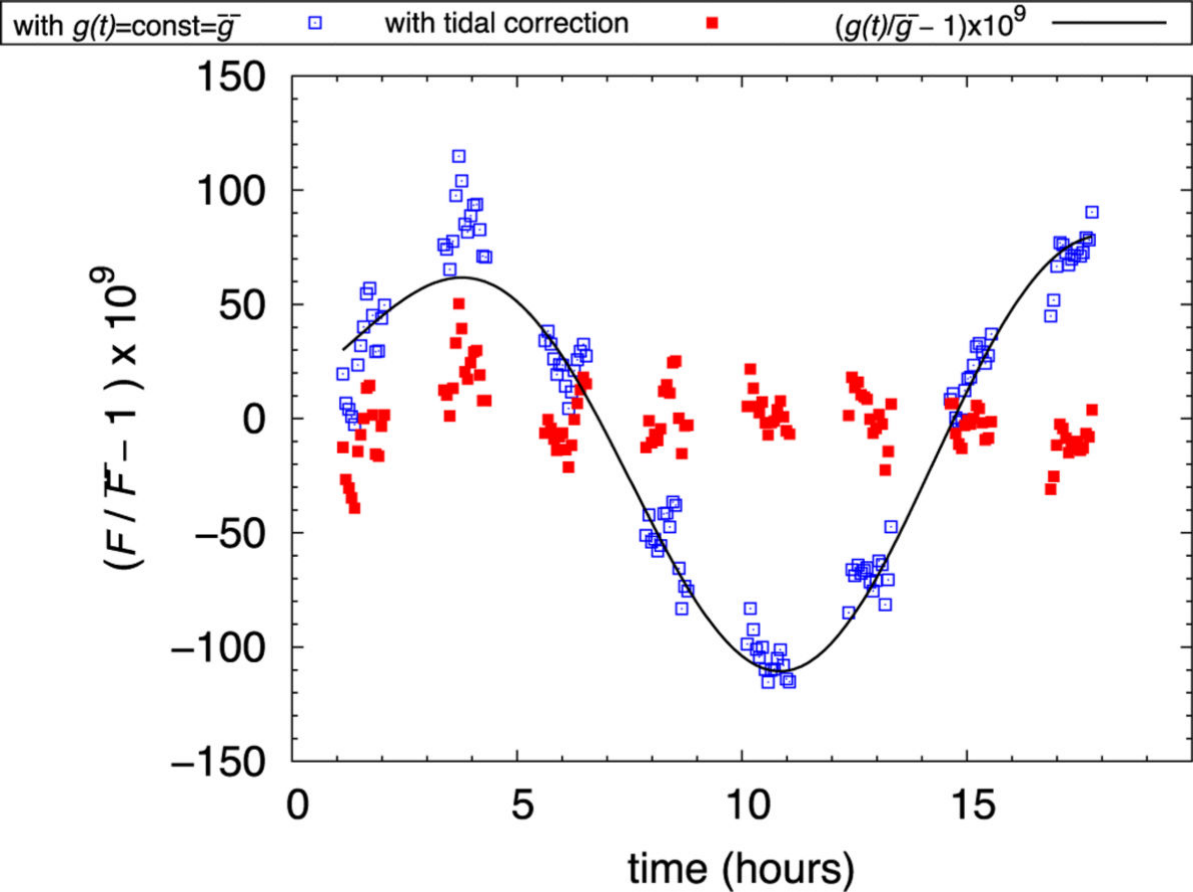
Measurements in force mode. The blue squares represent the force measurements obtained from the difference in voltage between mass on and off, the calibration values of the mass *m*, the resistance *R*, and assuming a constant gravity acceleration *g*. The red squares are the force measurements after tidal, pressure, and polar motion corrections for *g* have been applied.

First measurements of the local acceleration of gravity, vertical, and horizontal gravity gradients of the NIST laboratory 218/E024 have been made. A value for g of 9.801 031 394(43) ms^−2^ at the center of the mass on the balance has been determined in September 2015. This value is in good agreement with the previously reported value of 9.801 031 402(43) ms^−2^ from measurements taken in July 2013.^45^ The air pressure, tidal, and polar motion corrections are determined at each measurement of the flux integral in the force mode (Fig. 13).

An additional test was conducted to address the concern as to whether the flux produced by the current carrying coil will change the magnetization of the Sm_2_Co_17_ magnet rings. This would lead to a difference in the flux integral between the velocity and the force modes. In the force mode, the flux integral can be affected by the current *I* flowing in the coil and can be modeled similarly to the expressions developed in prior work:^14,16^

(16) $$Bl(I)=Bl(0)\left(1+\alpha I+\beta I^{2}\right),$$

where *Bl*(0) is the flux integral when no current is flowing in the moving coil, as in the velocity mode.

In force mode, two types of weighings are performed: mass off (subscript _off_) and mass on (subscript _on_). With the previous parameterization of the flux integral, the equation in the force mode can be written as,

(17) $$mg=Bl\left(I_{\text{off}\;}\right)I_{\text{off}\;}-Bl\left(I_{\text{on}\;}\right)I_{\text{on}\;}.$$

From the measured values of *I*_off_ and *I*_on_, the average value of the current and the current magnitude can be obtained using

(18) $$\bar{I}=\frac{1}{2}\left(I_{\text{off}}+I_{\text{on}}\right)\;\text{and}\;\Delta I=\frac{1}{2}\left(I_{\text{off}}-I_{\text{on}}\right).$$

In this campaign of measurements, *Ī* and ∆*I* are on average 1.5 μA and 6.9 mA, respectively. Applying this variable transformation of the current yields

(19) $$mg=2Bl(0)\Delta I\left(1+2\alpha \bar{I}+\beta \left(3{\bar{I}}^{2}+\Delta I^{2}\right)\right).$$

The value of *α* was measured by adding masses to the counter-mass and comparing results measured with this additional load to normal measurements. A 0.5 kg stainless steel (SS) and a combined stack of 1.5 kg SS masses along with the 1 kg Pt-Ir mass, were used to determine the quadratic term *β*. We obtained a value of −2.351(0.003) × 10^−6^ mA^−1^ for α and 0.368(0.323) × 10^−9^ mA^−2^ for *β*. The uncertainty contribution of the magnet non-linearity is dominated by our knowledge of the quadratic coefficient, *β*. At this time, the entry in the uncertainty budget for this item is 15.4 × 10^−9^.

## VI. PLANCK CONSTANT DETERMINATION

Careful determination of the correction terms and analysis of the Type A and Type B uncertainties lead to

(20) $$h/h_{90}-1=148\times 10^{-9}\pm 34\times 10^{-9}.$$

Therefore, the Planck constant value in SI units is

(21) $$h=6.626 069 83(22)\times 10^{-34}\text{Js}.$$

**FIG. 14.**
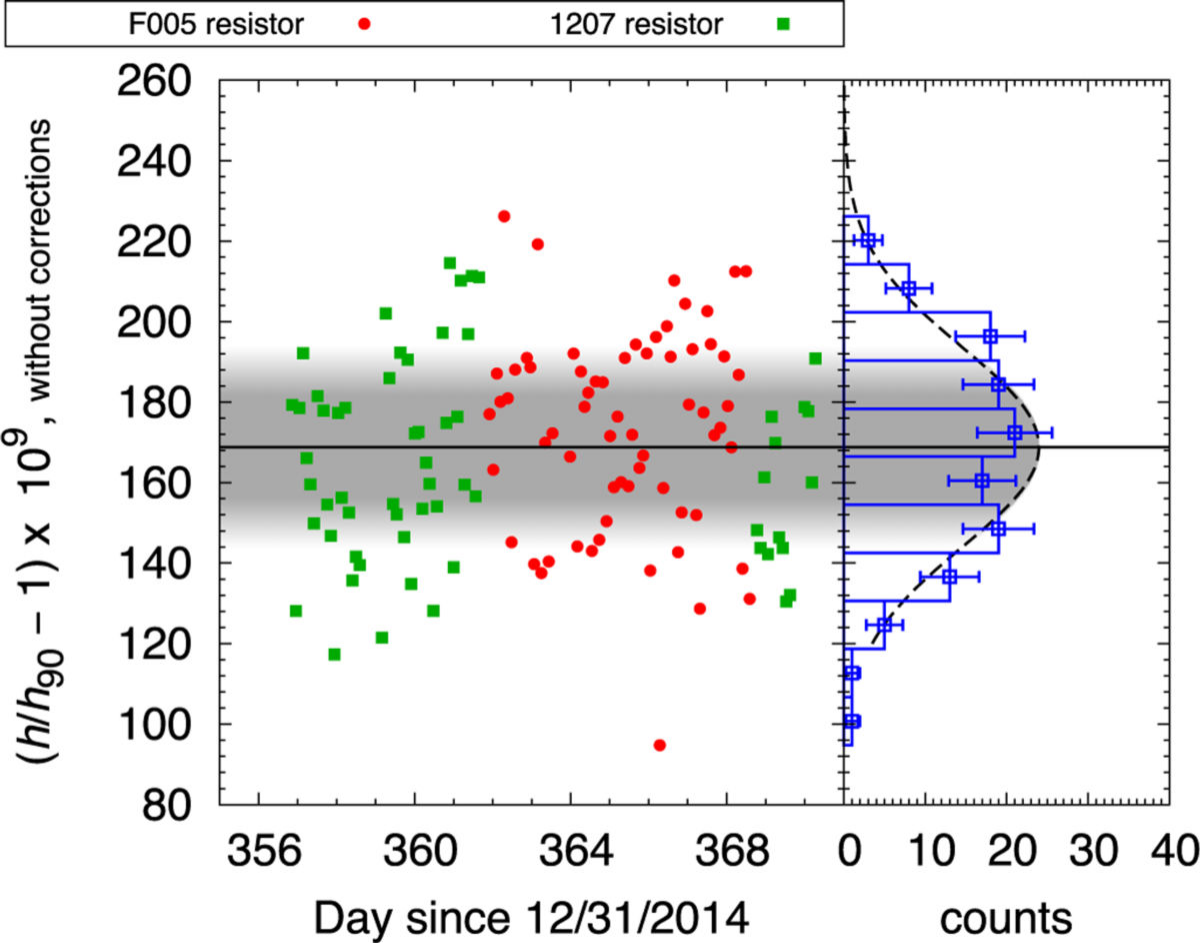
Measured values of the Planck constant, that are corrected only for the Abbe offset bias as well as air pressure, tidal, and polar motion. Each point in the left graph represents a determination of the Planck constant value, where in green it was determined with an oil bath resistor 1207 and in red with an air bath resistor F005. The right graph shows a histogram of all values of the Planck constant collected in this campaign. The error bars in the histogram denote the 1-sigma statistical uncertainty assuming a Poisson distribution of the frequency in each bin.

The number in parentheses denotes the one-sigma uncertainty in the last two digits. We have discussed in this article three components in the uncertainty budget of Table III. The statistical uncertainty is the sample standard deviation of the data presented in Fig. 14. The standard deviation of the mean is 2.3 × 10^−9^. As this is the first measurement campaign with NIST-4 watt balance, we are just starting to establish the nature of the statistical noise and checking if the measured Planck values stem from a normal and stationary statistical distribution. If this can be shown, the smaller standard deviation of the mean can be used for the statistical uncertainty, hopefully in a future publication. The alignment uncertainty is the combined uncertainty from Tables I and II plus the correlation terms. The magnetic field uncertainty is discussed in the previous section. The electrical uncertainty consists of uncertainties in the resistance and voltage metrology. This line item also includes leakage currents ground and time dependent leakage across the coil. The uncertainty labeled mathematical stems from fitting the profile of the flux integral as a function of *z*. The result can be influenced to a small degree by using orthogonal polynomials of different orders, different types and by changing the data range used in the fit. The uncertainty incurred by the choices made in the analysis presented here is estimated to be 5 × 10^−9^. The category balance mechanics estimates a potential bias created by the knife edge in the force mode. As stated above, this uncertainty is created by the fact that the knife edge excursions for loading and unloading the test mass are different. The effect was estimated by performing weighing after forcing the balance to go through similar excursions. The velocity uncertainty combines the uncertainties associated to the frequency reference, refractive index, beam shear, diffraction, frequency leakage, synchronization of voltage and velocity measurements, time interval analyzer timing, and photo-receiver jitters.

**FIG. 15.**
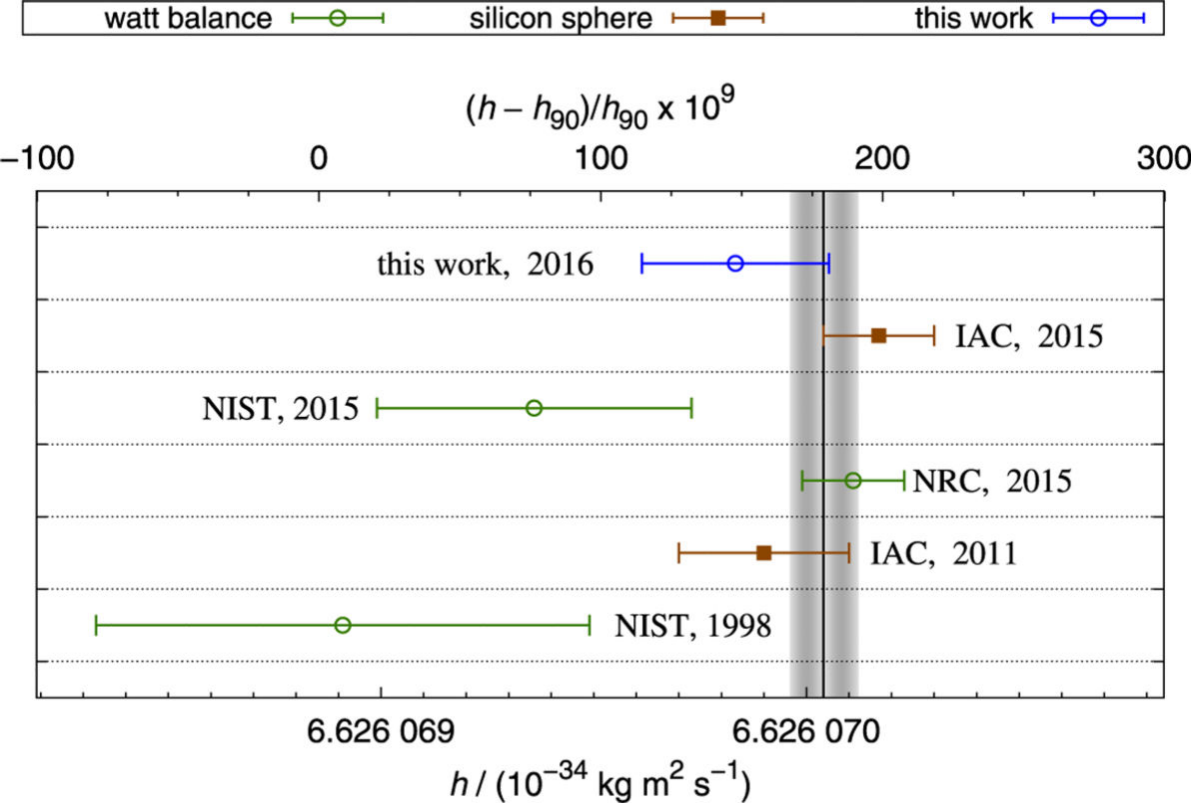
Determination of the Planck constant values used for the 2014 CODATA adjustment, marked with the vertical black line. The result of this work is less than 1-sigma away from all recent determinations of the Planck constant. The labels NRC, IAC, and NIST refer to published results.^46–50^ The dates indicate the year of publication.

Figure 15 shows the result of this work in comparison to other precision determinations of the Planck constant. The value obtained with NIST-4 agrees well with the results published by the International Avogadro Coordination (IAC) and the result measured with the watt balance at the National Research Council (NRC) in Canada.

## VII. EPILOGUE-2016

The present system of units has seven base quantities: time, length, mass, electric current, thermodynamic temperature, amount of substance, and luminous intensity. In particular, the unit of mass, the kilogram is still defined by an artifact, the international prototype of the kilogram (IPK) rather than a fundamental constant of nature. The current SI definition of the unit of mass is: “The kilogram is the unit of mass; it is equal to the mass of the international prototype of the kilogram.” Plans are under way to reform the SI in 2018, to a more fundamental system of units not based on artifacts. The new SI will also have seven base quantities: frequency, velocity, action, electric charge, heat capacity, amount of substance, and luminous flux/power. The specific reference quantities will be the exact values of a set of defining constants: the ground-state hyperfine splitting of the cesium-133 atom ∆*ν*(^133^Cs), the speed of light *c*, the Planck constant *h*, the elementary charge *e*, the Boltzmann constant *k*, the Avogadro constant *N*_A_, and the luminous efficacy *K*_cd_. In the new SI, the unit of mass can be realized using a watt balance from the fixed value of the Planck constant *h*^51,52^ by rewriting Eq. (5) as

(22) $$m=\frac{pn^{2}}{4}\frac{f_{U}^{2}}{g\nu_{z}}h,$$

where all quantities are based on their determined values in SI units.

In the first 3 months of 2016, we are realizing the unit of mass as part of the pilot study led by the BIPM, a preparatory step toward the redefinition of the kilogram. After the pilot study, some upgrades to the apparatus will be implemented. The off-the-shelf polarizing beam splitters used for the main interferometers have a good extinction ratio on only two faces, which causes some frequency leakage in the arm of the interferometer and will be replaced by custom-made beam splitters with a better extinction ratio. A vacuum mass transfer system has been developed consisting of a load lock, mass exchange points and vacuum transfer arms to transfer the standard mass from the NIST-4 apparatus, which will be the primary realization of the kilogram, to the magnetic levitation apparatus, an adjacent experiment to the watt balance, which will make the link between the vacuum standard mass to the air test masses. We plan to have a more precise value of the Planck constant published before July 2017, the deadline set by CODATA for data that will be used for the final adjustment of the fundamental reference constants. After redefinition of the unit of mass, the watt balance NIST-4 will be the primary realization of the kilogram in the United States.

## Figures and Tables

**TABLE I.** Biases in the velocity measurement introduced by misalignment. The first four elements come from the misalignment *α*_*x*_ and *α*_*y*_ from vertical of the laser beams about the *x* and *y*-axis respectively. The subscript *i* denotes the number of the heterodyne interferometer that determine the vertical velocity *ν*_*z*_. The angle *α*_*i*_ in the third row is calculated as *α*_*yi*_sin*A*_*i*_+*α*_*xi*_cos*A*_*i*_, where *A*_*i*_ is the angular location of the *i* th interferometer. The last two elements represent the Abbe offset errors. The total standard uncertainty is the square root of the sum of the squared individual uncertainty components.

Description	Term	1st factor	2nd factor	Product (nJ s/J s)
Motion of retroreflectors in *x*	$\frac{1}{3}\sum_{i=1}^{3}-\alpha_{yi}\left(\nu_{x}/\nu_{z}\right)$	45.44 × 10^−6^	2.21 × 10^−4^	10.05± 3.65
Motion of retroreflectors in *y*	$\frac{1}{3}\sum_{i=1}^{3}\alpha_{xi}\left(\nu_{y}/\nu_{z}\right)$	−13.96 × 10^−6^	−1.17 × 10^−4^	1.64 ± 2.37
Motion of retroreflectors about *z*	$\frac{1}{3}\sum_{i=1}^{3}\left(\alpha_{i}\right)\left(R\omega_{z}/\nu_{z}\right)$	−4.74 × 10^−5^	1.08 × 10^−4^	−5.09± 2.84
Cosine attenuation of *ν_z_*	$\frac{1}{3}\sum_{i=1}^{3}\left(\frac{-\alpha_{xi}^{2}}{2}+\frac{-\alpha_{yi}^{2}}{2}\right)\left(\nu_{z}/\nu_{z}\right)$	−2.68 × 10^−9^	1	−2.68± 1.59
Optical center deviation in *x*	$d_{x}\left(\omega_{y}/\nu_{z}\right)$	0.00 × 10^−3^ mm	4.00 × 10^−7^ mm^−1^	0.00 ± 0.74
Optical center deviation in *y*	$d_{y}\left(\omega_{x}/\nu_{z}\right)$	0.00 × 10^−3^ mm	2.39 × 10^−7^ mm^−1^	0.00 ± 0.38
			Total	3.99± 5.48

**TABLE II.** Biases in the voltage measurement due to the six degrees of freedom of the coil. The total standard uncertainty is the square root of the sum of the squared individual uncertainty components.

Description	Term	1st factor	2nd factor	Product (nJ s/J s)
Derivative of flux w.r.t. *x*	$\left(F_{x}/F_{z}\right)\left(\nu_{x}/\nu_{z}\right)$	−4.78×10^−6^	2.21×10^−4^	−1.06 ±0.24
Derivative of flux w.r.t. *y*	$\left(F_{y}/F_{z}\right)\left(\nu_{y}/\nu_{z}\right)$	1.67×10^−6^	−1.17×10^−4^	−0.19±0.35
Derivative of flux w.r.t. *θ_x_*	$\left(\tau_{x}/F_{z}\right)\left(\omega_{x}/\nu_{z}\right)$	1.14×10^−3^ mm	2.39×10^−7^ mm^−1^	0.27±0.04
Derivative of flux w.r.t. *θ_y_*	$\left(\tau_{y}/F_{z}\right)\left(\omega_{y}/\nu_{z}\right)$	3.91×10^−4^ mm	4.00×10^−7^ mm^−1^	0.15±0.04
Derivative of flux w.r.t. *θ_z_*	$\left(\tau_{z}/F_{z}\right)\left(\omega_{z}/\nu_{z}\right)$	−8.27×10^−5^ mm	5.0×10^−7^ mm^−1^	−0.04±2.23
			Total	ࢤ0.87±2.27

**TABLE III.** Sources of uncertainty in this measurement of *h*. All entries are relative standard uncertainties (*k* =1).

Source	Uncertainty (10^−9^)
Statistical	24.9
Magnetic field	15.4
Electrical	10.9
Alignment	6.5
Mass metrology	6.3
Mathematical	5.0
Balance mechanics	5.0
Local acceleration, *g*	4.4
Velocity	1.7
Total relative uncertainty	33.6
